# Synchronous excitation in the superficial and deep layers of the medial entorhinal cortex precedes early sharp waves in the neonatal rat hippocampus

**DOI:** 10.3389/fncel.2024.1403073

**Published:** 2024-04-26

**Authors:** Dmitrii Shipkov, Azat Nasretdinov, Roustem Khazipov, Guzel Valeeva

**Affiliations:** ^1^Laboratory of Neurobiology, Institute of Fundamental Medicine and Biology, Kazan Federal University, Kazan, Russia; ^2^INMED - INSERM, Aix-Marseille University, Marseille, France

**Keywords:** entorhinal cortex, hippocampus, sharp wave, neonatal rat, local field potentials, multiple unit activity, current-source density

## Abstract

Early Sharp Waves (eSPWs) are the earliest pattern of network activity in the developing hippocampus of neonatal rodents. eSPWs were originally considered to be an immature prototype of adult SPWs, which are spontaneous top-down hippocampal events that are self-generated in the hippocampal circuitry. However, recent studies have shifted this paradigm to a bottom-up model of eSPW genesis, in which eSPWs are primarily driven by the inputs from the layers 2/3 of the medial entorhinal cortex (MEC). A hallmark of the adult SPWs is the relay of information from the CA1 hippocampus to target structures, including deep layers of the EC. Whether and how deep layers of the MEC are activated during eSPWs in the neonates remains elusive. In this study, we investigated activity in layer 5 of the MEC of neonatal rat pups during eSPWs using silicone probe recordings from the MEC and CA1 hippocampus. We found that neurons in deep and superficial layers of the MEC fire synchronously during MEC sharp potentials, and that neuronal firing in both superficial and deep layers of the MEC precedes the activation of CA1 neurons during eSPWs. Thus, the sequence of activation of CA1 hippocampal neurons and deep EC neurons during sharp waves reverses during development, from a lead of deep EC neurons during eSPWs in neonates to a lead of CA1 neurons during adult SPWs. These findings suggest another important difference in the generative mechanisms and possible functional roles of eSPWs compared to adult SPWs.

## 1 Introduction

Early Sharp Waves (eSPWs) are the earliest network activity pattern in the developing hippocampus of neonatal rodents (Leinekugel et al., 2002; Karlsson et al., 2006; Mohns et al., 2007; Marguet et al., 2015; Unichenko et al., 2015; Valeeva et al., 2019a,b; Murata and Colonnese, 2020; Graf et al., 2021; Cossart and Khazipov, 2022; Pochinok et al., 2024). The originally proposed paradigm implies that eSPWs are an immature prototype of adult SPWs, except that eSPWs lack the high frequency oscillations (ripples, Rs) that are characteristic of adult SPWs and appear after P10 (Leinekugel et al., 2002; Buhl and Buzsaki, 2005; Pochinok et al., 2024), and that eSPWs are reliably triggered by myoclonic movements, presumably via sensory feedback from movements (Karlsson et al., 2006; Mohns and Blumberg, 2010; Valeeva et al., 2019a,b). In addition, eSPWs, but not adult SPW-Rs, are triggered by somatosensory stimulation (Brankack and Buzsaki, 1986; Bellistri et al., 2013; Gainutdinov et al., 2023). However, recent studies have shifted this paradigm, suggesting that eSPW network mechanisms differ from adult SPW-Rs. In fact, adult SPW-Rs are endogenous, self-generated events in the hippocampal circuitry and are considered to be top-down events that transfer information to target brain regions and support the consolidation of memories acquired during exploration (Buzsaki, 2015). In contrast, eSPWs are generated in a bottom-up fashion in the entorhinal-hippocampal circuit and are primarily driven by inputs from layers 2/3 of the medial entorhinal cortex (MEC) (Valeeva et al., 2019a,b). Synchronized firing of neurons in the superficial layers of the MEC is associated with so-called sharp potentials (MEC-SPs), which precede hippocampal eSPWs and are triggered by physiological myoclonic movements (Valeeva et al., 2019a,b). Thus, the entorhinal-hippocampal MEC-SP – eSPW complexes are embedded within the large-scale network activated during twitches and startles, and which likely involves sensory feedback from myoclonic movements conveyed from somatosensory cortex to MEC and further to hippocampus (Karlsson et al., 2006; Mohns and Blumberg, 2010; Valeeva et al., 2019a,b; Gainutdinov et al., 2023). It has been suggested that entorhinal-hippocampal MEC-SP – eSPW complexes underlie the sequential, activity-dependent maturation of connections between the MEC and the hippocampus and within the hippocampal circuitry (Donato et al., 2017; Valeeva et al., 2019a,b; Cossart and Khazipov, 2022).

A hallmark of adult SPW-ripples is the relay of information from the CA1 hippocampus to target structures, including L5 of the MEC, with further transfer of transiently stored hippocampal information to long-term engrams in neocortical networks (Buzsaki, 1986; Siapas and Wilson, 1998; Girardeau et al., 2009; Nakashiba et al., 2009; Buzsaki, 2015; Squire et al., 2015). During SPW-Rs, MEC L5 neurons are activated following CA1 pyramidal cells by direct monosynaptic CA1 to L5 inputs or via intermediate activation of subicular neurons (Chrobak and Buzsaki, 1994, 1996; Isomura et al., 2006; Roth et al., 2016; Rozov et al., 2020). Whether and how deep layers of MEC are activated during eSPWs in the neonates, and whether CA1 inputs to MEC drive L5 neurons similarly to adult SPW-Rs, remains elusive. The latter scenario is supported by studies using intact limbic structures preparation from neonatal rats *in vitro*, in which kainate-induced hippocampal seizures propagated to the EC suggesting the existence of functional connections from the hippocampus to the EC as early as P4 (Khalilov et al., 1999). On the other hand, hippocampal CA3-generated giant depolarizing potentials (GDPs) (Ben-Ari et al., 1989), which have been considered as an *in vitro* counterpart of eSPWs (Leinekugel et al., 2002; Ben Ari et al., 2007; Griguoli and Cherubini, 2017), do not propagate to the EC (Khalilov et al., 1999; Namiki et al., 2013). Furthermore, the presence of spontaneous waves of activity involving both superficial and deep EC layers in neonatal mouse brain slices suggests the existence of intracortical mechanisms for horizontal and vertical synchronization in the developing EC network (Sheroziya et al., 2009; Namiki et al., 2013; Unichenko et al., 2015). Here, we investigated how activity in deep EC layers is organized in relation to MEC-SPs and hippocampal eSPWs *in vivo*. We found that neurons in deep and superficial MEC layers fire synchronously during MEC-SPs, and that neuronal firing in both superficial and deep EC layers precedes the activation of CA1 neurons during eSPWs. Thus, the sequence of activation of hippocampal CA1 neurons and deep EC neurons during sharp waves reverses during development, from a lead of deep EC neurons during eSPWs in neonates to a lead of CA1 neurons during adult SPWs. Our findings suggest another important difference between eSPWs and adult SPWs, supporting the paradigm shift in views of the function of the developing entorhinal-hippocampal network.

## 2 Materials and methods

### 2.1 Ethical approval

The animal experiments were carried out in compliance with the ARRIVE guidelines. Animal care and procedures were in accordance with EU Directive 2010/63/EU for animal experiments, and all animal-use protocols were approved by the French National Institute of Health and Medical Research (APAFIS #16992-2020070612319346 v2) and the Local Ethical Committee of Kazan Federal University (#24/22.09.2020).

### 2.2 Animal preparation

**Figure 1:**
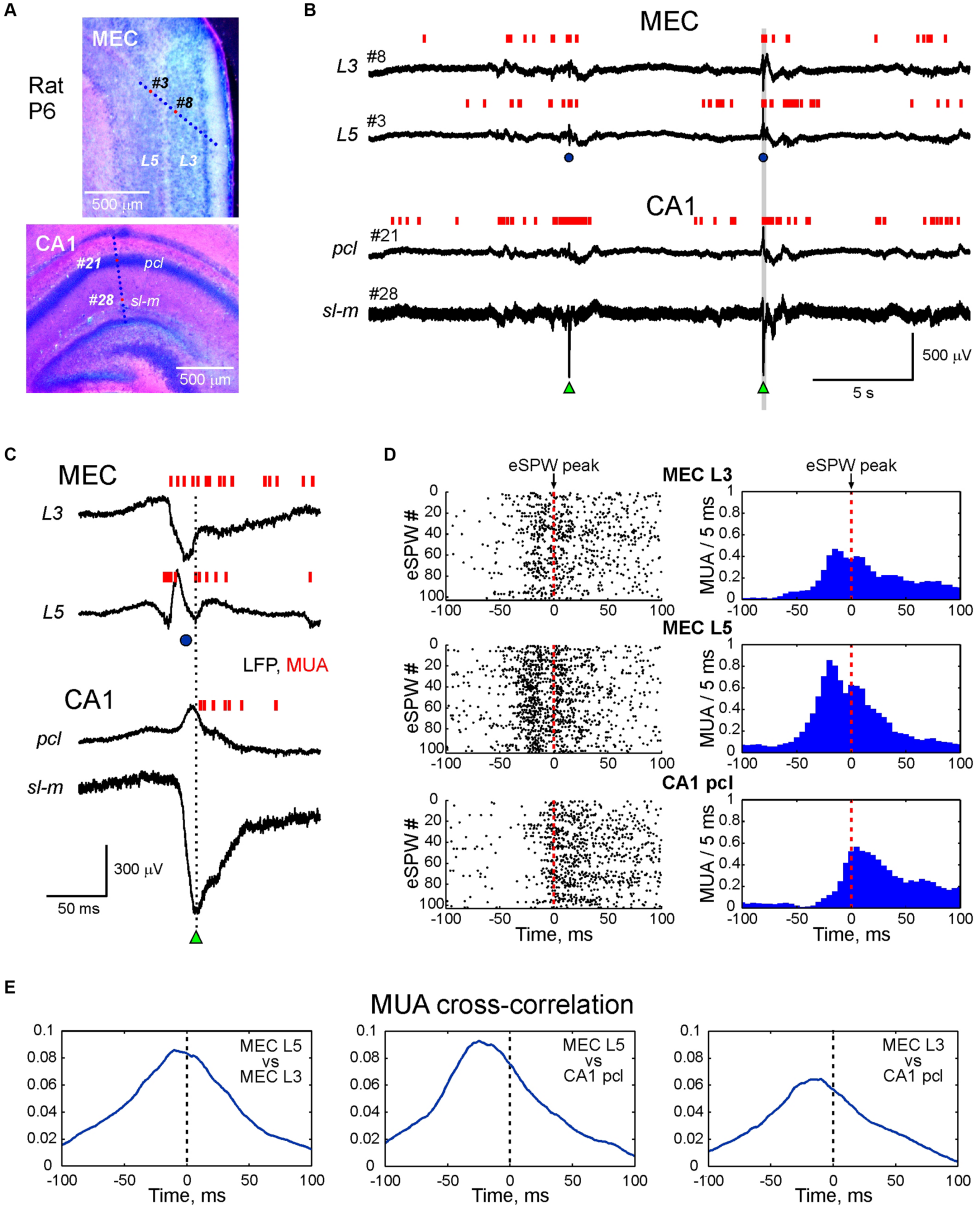
Activity bursts in MEC L3 and L5 associated with sharp potentials precede early hippocampal sharp waves in the neonatal rat. **(A)** Recording sites of multielectrode arrays overlaid on a cresyl violet stained sagittal MEC slice (top panel) and coronal hippocampal slice (bottom panel) in a P6 rat pup. **(B)** Simultaneous LFP recordings in MEC L5 and L3 (recording sites # 3 and # 8 on top panel **A**), and hippocampal CA1 pyramidal cell layer (pcl) and stratum lacunosum-moleculare (sl-m) (recording sites # 21 and # 28 on bottom panel **A**). Multiple unit activity (MUA) is represented by vertical red bars. Hippocampal early sharp waves (eSPWs) are indicated by green triangles, sharp potentials in MEC (MEC-SPs) are indicated by blue circles. **(C)** An example of MEC L3/L5 burst and eSPW complex from panel **(B)** (highlighted in a gray box) on expanded time scale. **(D)** eSPW-triggered raster plots (left) and PETHs (right) for MUA in MEC L5 and L3, and in CA1 pcl. **(E)** MUA cross-correlograms in MEC L5 vs. MEC L3 (left), MEC L5 vs. CA1 (middle) and MEC L3 vs. CA1 (right) during peri-eSPW epochs (*n* = 102 eSPWs).

Wistar rats of both sexes from postnatal days (P) 4–7 were used. Preparation of the animals for head-restrained recordings was performed under isoflurane (1.5–2.5%) anesthesia. The skull of the animal was cleaned of skin and periosteum using Hemostab Al solution (Omega Dent, Russia), dried and covered with a thin layer of cyanacrylamide glue and self-curing acrylic denture repair material (Meliodent RR, Kulzer, GmbH, Germany), leaving the surface of left parietal bone open. A metal ring was fixed to the skull by dental acrylic material and via ball-joint to a magnetic stand. The wound was treated with bupivacaine (5%). The animal was then wrapped in a cotton and warmed at a thermal pad (37°C, Warner Instr., United States) and left for an hour to recover from anesthesia. None of the animals showed any signs of discomfort or pain (as evidenced by the absence of prolonged and excessive movements) during the recordings. Extracellular recordings of local field potentials (LFP) and multiple unit activity (MUA) were performed along the CA1—dentate gyrus axis of the dorsal hippocampus and the dorsal part of MEC in the left hemisphere (Figure 1) using 16-channel linear silicon probes with 50 μm separation distance between the electrodes (NeuroNexus, United States). Of note, eSPWs are expressed and highly synchronized along the longitudinal axis of the hippocampus and bilaterally in neonatal rat pups (Valeeva et al., 2019a,b, 2020). Silicon probes were placed using stereotaxic coordinates provided by an atlas of the postnatal rat brain (Khazipov et al., 2015). For hippocampal recordings, electrodes were placed at—2.1 mm posterior and 1.35 mm lateral from bregma at depth of 2,300–2,600 μm; the lateral-medial angle from the horizontal plane 75°. For MEC recordings, electrodes were placed as in (Quilichini et al., 2010), at 1.6 mm anterior and 3.7 mm lateral from lambda at depth 3,000–3,600 μm; the anterior–posterior angle from the horizontal plane 45°. In a subset of animals, electrodes were placed into MEC along the MEC layers as in Valeeva et al. (2019a,b) at the medial-lateral angle from the horizontal plane 75° (Supplementary Figure S3). For the histological reconstruction of electrode tracks, electrodes were coated with ethanol-dissolved DiI (Sigma-Aldrich, United States). A сhlorided silver wire, placed in the neocortex, served as a ground electrode. Signals from extracellular recordings were amplified and filtered (10,000X; 0.15 Hz–9 kHz) using Digital Lynx SX amplifier (Neuralynx, United States), digitized at 16–32 kHz. From one to 2 h of spontaneous activity were recorded from each animal.

### 2.3 Histology

After recordings the animals were deeply anaesthetized with isoflurane (5%), the brains were removed and left for fixation in 4% paraformaldehyde for 2 days at room temperature. Then the brains were rinsed in PBS and mounted in agar blocks. Brains were cut into 100 μm-thick slices using Vibratome (Thermo Fisher Scientific, MA, United States) in two steps. First, coronal slices were cut in rostral-caudal direction to obtain full DiI track of the hippocampal probe. Then the two hemispheres in remaining block were separated, and sagittal slices were prepared from the left hemisphere to reveal the DiI track of the MEC probe. The location of the silicone probe in hippocampus and enthorhinal cortex was assessed through identification of the DiI track in serial 100-μm-thick sagittal sections (Supplementary Figures S1, S2). Then DiI tracks were overlaid on the microphotographs of brain slices after cresyl violet staining. In hippocampal recordings, electrode location was verified by the highest MUA rate in CA1 stratum pyramidale. In MEC recordings, electrode location was adjusted according to MEC-SP LFP reversal around L4.

### 2.4 Data analysis

Raw data were preprocessed using custom-written functions in MATLAB (MathWorks, United States). Hippocampal eSPWs were detected from down-sampled (1,000 Hz), bandpass filtered (3–100 Hz, Chebyshev type 2 Filter) LFPs. All troughs greater than 2–4 SD from the least active 100 s long epoch through the entire record were first detected from the channel located in the stratum lacunosum–moleculare (*sl-m*) and their peak negativity was taken as time = 0 for further analysis. Independently, LFP peaks exceeding 1–3 SD were similarly detected from the CA1 pyramidal cell layer (*pcl*). Negative *sl-m* events with a half-width ≤ 65 ms co-occurring with positive *pcl* peaks in the within ±50 ms time window were considered as eSPWs. To discard movement artifacts, LFP segments from −0.5 s to 1 s around the eSPW peak negativity for each channel were visually inspected. MEC sharp potentials (MEC-SPs) were detected from the channel displaying maximal negativity within a time window from −0.5 s to 0.5 s around the eSPW similarly to the procedure of eSPWs detection described above. Current-source density (CSD) analysis across MEC depth was performed on averaged MEC-SPs according to a differential scheme for second derivative and smoothed with a triangular kernel of length 4 (Freeman and Nicholson, 1975).

For multiple unit activity (MUA) analysis, raw LFP recordings were band-pass filtered in the range of 250–4,000 Hz (Daubechies wavelet filter). Action potentials were detected as negative peaks below 4 SD of the least active 100 s long epoch over the entire recording. Peri-event time histograms (PETHs) were calculated for MUA in 1 ms bins relative to the eSPW times followed by smoothing with the 50 ms window sliding average filter. MUA cross-correlograms were calculated in 1 ms bins for peri-eSPW epochs of [−50 + 100] ms relative to the eSPW times followed by smoothing with the 30 ms window sliding average filter.

### 2.5 Statistical analysis

Statistical analysis was performed using the MATLAB Statistics toolbox. Group comparisons were performed using the two-sided Wilcoxon rank sum and Wilcoxon signed rank tests. Unless otherwise noted, group data are presented as median (Q1–Q3).

## 3 Results

In the present study, we explored the dynamics of neuronal network activity across layers of the MEC in association with hippocampal eSPWs in neonatal rats. For this purpose, we performed simultaneous recordings of LFPs and multiple unit activity (MUA) from the dorsal CA1 hippocampus and MEC in non-anaesthetized, head-restrained postnatal day [P] 4–7 rats. The location of the recording sites was determined during *post-hoc* analysis of the DiI electrode traces in coronal slices for hippocampal recordings and sagittal slices for MEC recordings with silicone probes inserted across the MEC layers (*n* = 18 rats) (Figure 1A; Supplementary Figures S1, S2) or parallel to the MEC layers (*n* = 10 rats; Supplementary Figure S3). Consistent with previous studies, activity in the MEC and hippocampus was characterized by discontinuous temporal organization and complexes of intermittent eSPWs in the hippocampus occurring at a frequency of 1.4 (0.9–1.8) per minute (*n* = 18 rats), preceded by bursts of MEC activity often associated with large amplitude sharp potentials (MEC-SPs) (Leinekugel et al., 2002; Karlsson et al., 2006; Mohns et al., 2007; Marguet et al., 2015; Unichenko et al., 2015; Valeeva et al., 2019a,b; Murata and Colonnese, 2020; Graf et al., 2021; Pochinok et al., 2024). Example recordings from L3 and L5 of MEC and CA1 hippocampus (*pcl* and *sl-m*) are shown in Figures 1B,C. eSPWs were characterized by negativity below the CA1 pyramidal cell layer and polarity reversal at the *pcl*, whereas MEC-SPs were associated with a negative sharp potential in superficial MEC layers 2 and 3 and polarity reversal at the level of L4 (see also below) (Figure 1C). We then examined how the activity of neurons in MEC and CA1 was modulated in relation to hippocampal eSPWs (Figures 1C–E). Raster plots and peri-event histograms of MUA in L3 and L5 of MEC and CA1 *pcl* aligned by eSPW peaks revealed a strong increase in MUA and co-activation of neurons in deep and superficial MEC layers, preceding the activation of CA1 neurons (Figure 1D). This was further confirmed by cross-correlation analysis of MUA in MEC L3 and L5 and in CA1 (Figure 1E).

**Figure 2:**
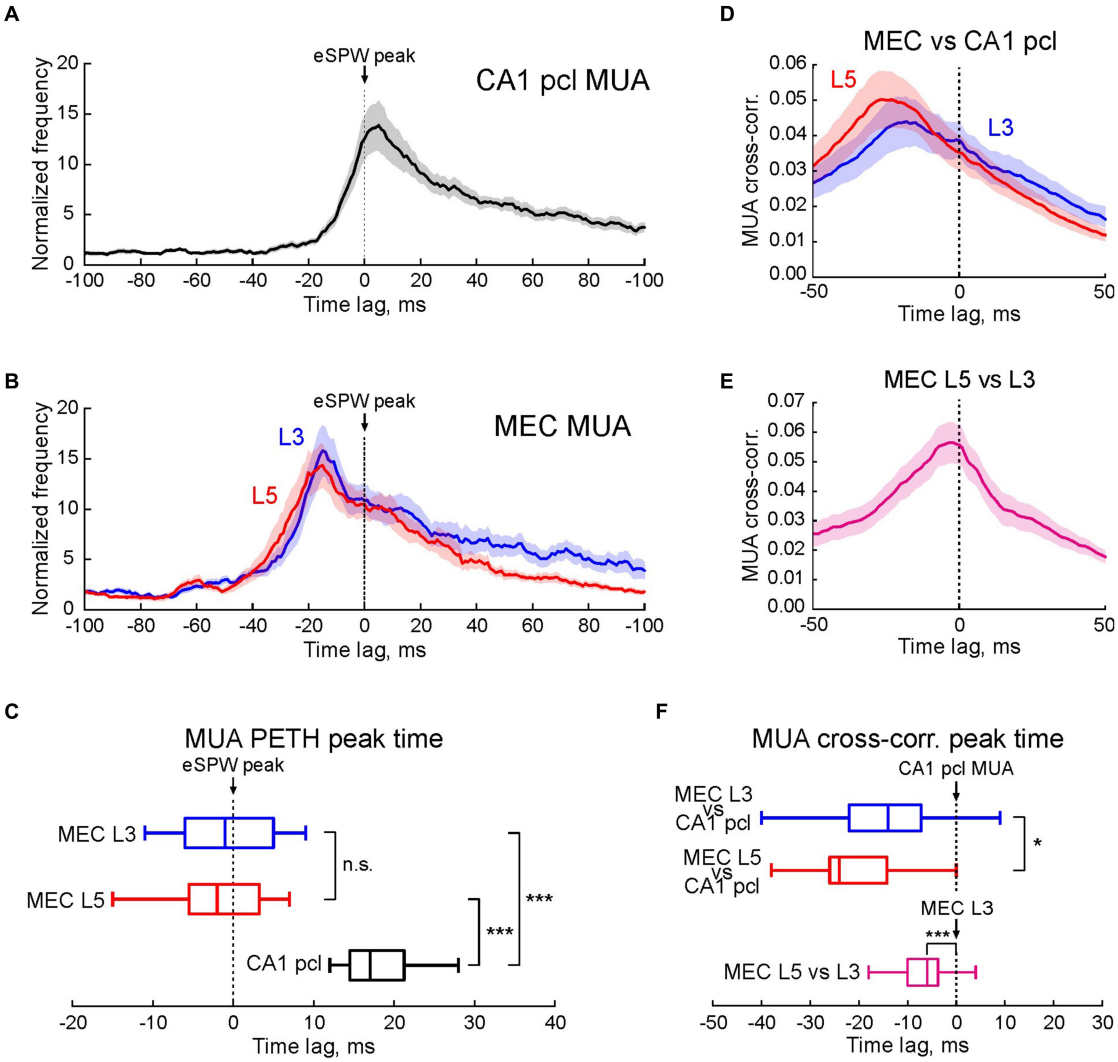
Multiple unit activity in MEC L3 and L5 and CA1 hippocampus in relation to early hippocampal sharp waves. **(A,B)** Average PETHs (mean ± SEM) of MUA in CA1 pcl **(A)** and MEC L3 and L5 **(B)** aligned to eSPW times and expressed as MUA frequency normalized to the baseline. **(C)** Horizontal boxplots of group data (center line, median; edges, Q1/Q3; whiskers, non-outlier extremes) for the time of peak firing of multiple units relatively to eSPWs (two-sided Wilcoxon rank sum test). ***p value < 0.001. **(D,E)** Average MUA cross-correlograms in MEC L3 and L5 vs. CA1 *pcl* **(D)**, and MEC L5 vs. MEC L3 **(E)** Shaded lines, SEM. **(F)** Boxplots of group data (center line, median; edges, Q1/Q3; whiskers, non-outlier extremes) for the time of cross-correlation peak of multiple units in MEC L3 and L5 vs. CA1 (top) and MEC L5 vs. MEC L3 (bottom) (two-sided Wilcoxon rank sum test). **(A–F)** Pooled data from 1,196 eSPWs recorded from *n* = 17 P5-7 rats. *p value < 0.05; ***p value < 0.001; n.s., non-significant.

We further analyzed neuronal activity in MEC L3 and L5 and CA1 hippocampus during eSPWs at the population level in a group of 17 P5-7 rats (Figure 2). Action potential firing in MEC L3 and L5, and in CA1 *pcl* increased during eSPWs (*n* = 17 rats; *p* < 0.001; Figures 2A,B; Supplementary Table S1). However, the peak activation of neurons in the hippocampus was significantly delayed compared to MEC, both in L3 and L5 (*n* = 17 rats; *p* < 0.001; Figure 2C; Supplementary Table S2). Notably, although the peak of MUA relatively to eSPWs in L5 had a tendency to precede that in L3, this difference was not significant, however. A ~ 20 ms delay in activation of units in the CA1 hippocampus from MEC units in L3 and L5 was also evident from cross-correlation analysis of all units detected in a time window of −50 to +100 ms relative to eSPWs (Figures 2D,F; Supplementary Tables S3, S4). Group data analysis of paired comparisons of MUA cross-correlation peaks revealed a significant precedence of MEC units (both in L3 and L5) relative to CA1 units (*n* = 17 rats; *p* < 0.05), and a short (by ~6 ms) but significant delay in activation of neurons in L3 from L5 (*n* = 17 rats; *p* < 0.001; Figures 2E,F; Supplementary Table S4).

**Figure 3:**
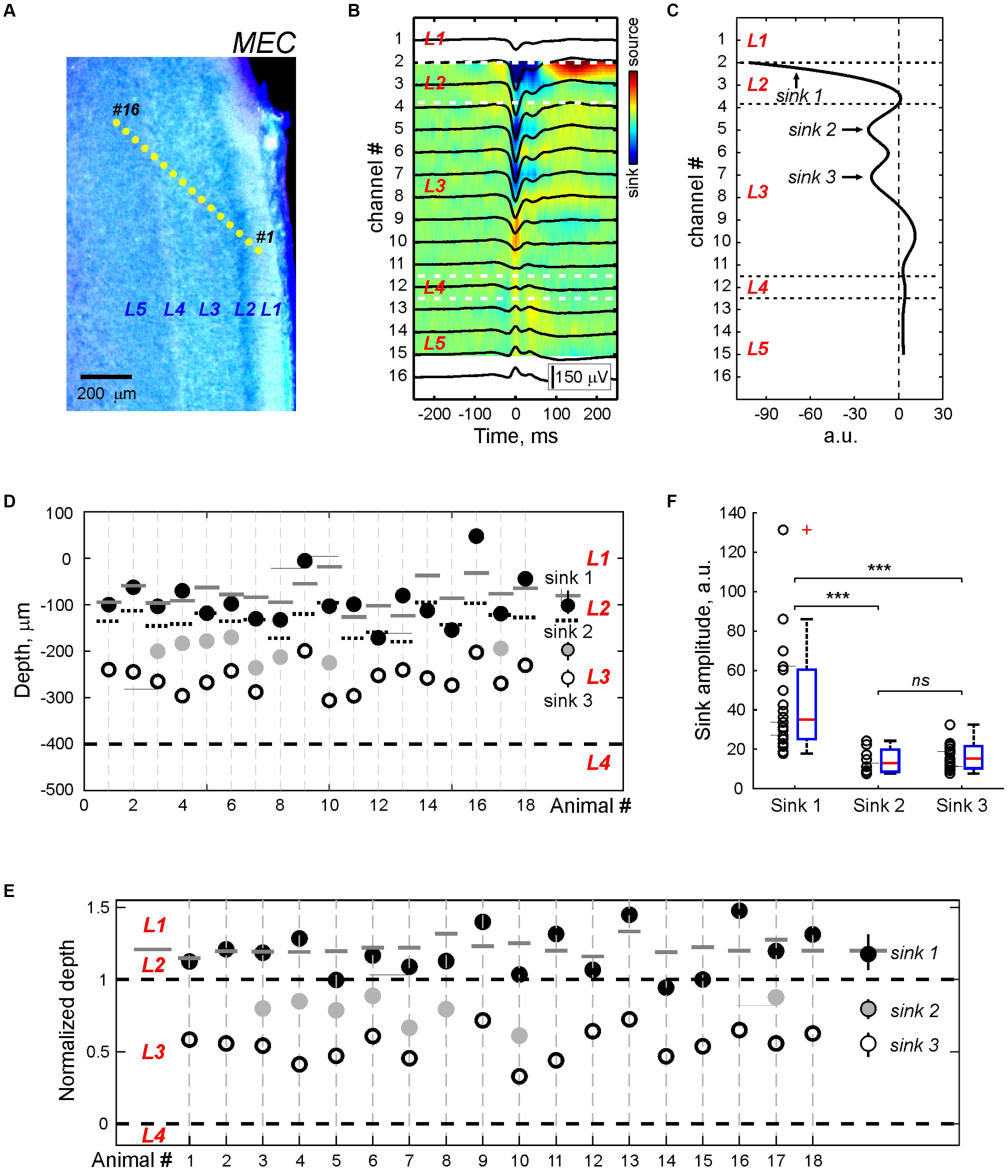
CSD profile of the MEC sharp potentials. **(A)** Recording sites of the multielectrode array overlaid on a cresyl violet stained sagittal MEC sections from a P5 rat. **(B)** Average local field potential (black traces) overlaid on the color-coded CSD map of the MEC-SP. **(C)** CSD profile at the peak of the MEC-SP shown on panel **(B)**. Note Sinks 1, 2, and 3 of the MEC-SPs in the superficial layers and a main source near L4. **(D,E)** Group data on the depth of the MEC-SP Sinks 1 (black circles), 2 (gray circles), and 3 (open circles) in relation to L3/L4 border (bottom dashed line) in absolute values **(D)** and normalized to the distance between L3/4 and L2/3 borders **(E)**. Right, group medians with Q1 and Q3. Pooled data were obtained from *n* = 18 P5–7 rats. On **(D)** and **(E)**, L2/L3 and L3/L4 borders are marked by black dashed lines, and L1/L2 border - by solid gray lines **(F)** Group data on the amplitude of current sinks associated with MEC-SPs. ***p value < 0.001; n.s., non-significant.

Next, we analyzed the LFP depth profile of MEC-SPs in 18 P5-7 animals with probe insertion across the MEC layers (Figure 3A). Consistent with previous studies *in vivo* (Valeeva et al., 2019a,b) and *in vitro* (Sheroziya et al., 2009; Namiki et al., 2013; Unichenko et al., 2015), MEC-SPs were characterized by negative LFP deflection in the superficial MEC layers (Figure 3B). In deep layers, MEC-SPs changed polarity to positive, with the reversal occurring around agranular L4. Similar LFP shapes of MEC-SPs with negativity in superficial layers and positivity in deep layers were also found at different cortical depths during “vertical” probe insertion along the MEC layers (Supplementary Figure S3), suggesting that this distinct LFP depth profile of MEC-SPs with polarity reversal around L4 may be useful for estimating electrode position in the MEC during recordings in neonatal rats. The CSD analysis of MEC-SPs revealed from two to three sinks distributed in layers 1, 2, and 3 (Figures 3B,C; Supplementary Table S5). The most superficial Sink 1 (Figures 3D,E; Supplementary Tables S5, S6) was usually located around L1/L2 border and had the largest amplitude (*n* = 18; *p* < 0.001; Figure 3F; Supplementary Table S7). The Sink 2 and Sink 3 were found within the superficial (close to L2) half of L3 and did not differ in amplitude from each other (Figures 3D–F; Supplementary Tables S5–S7). Thereby, the layerwise distribution of current sinks, reflecting areas of synaptic activation during MEC-SPs, matched the location of main external inputs to MEC (Witter et al., 2017). In addition, the most prominent Sink 1 was observed in MEC layers 1 and 2, containing the dendritic tufts of MEC neurons from all deeper layers (Canto et al., 2008).

## 4 Discussion

The main findings of the present study are that in neonatal rats, deep and superficial МEC neurons are co-activated during MEC-SPs, and that neuronal firing in both superficial and deep MEC layers precedes the activation of CA1 neurons during eSPWs. Thus, the sequence of activation of hippocampal CA1 neurons and deep MEC neurons during sharp waves changes during development from leadership of deep MEC neurons during eSPWs in neonates to leadership of CA1 neurons during SPW-Rs in adults. These results are consistent with the hypothesis that the network mechanisms underlying neonatal eSPWs are distinct from the mechanisms of adult SPWs generation, and support a paradigm shift from viewing of neonatal eSPWs as the prototype of adult SPW-Rs.

MEC-SP events, which precede hippocampal eSPWs and are triggered by myoclonic movements, have previously been described in the superficial layers of the MEC in neonatal rats (Valeeva et al., 2019a,b). In the present study, we investigated the spatiotemporal organization of MEC-SPs across cortical layers, including a description of their LFP and current-source density depth profiles and neuronal firing. We found that MEC-SPs are electronegative in superficial layers and positive in deep layers with a reversal around L4, and that their main sinks are distributed along the depth of superficial layers. The origin of the synaptic inputs that generate these sinks and drive MEC firing remains hypothetical for now. In adults, SPW-Rs cause activation of neurons in deep layers of the MEC either through direct connections from CA1 pyramidal cells or via the subiculum, whereas the activity of neurons in superficial MEC layers is weakly modulated by SPWs (Chrobak and Buzsaki, 1994, 1996). In contrast, during neonatal eSPWs, CA1 neurons are activated with a delay from neurons in MEC, and therefore the role of CA1-MEC connections in the generation of MEC-SPs is limited, at least in the initial part of MEC discharges. This raises the question of what are the generative network mechanisms of MEC-SPs? Because MEC-SPs are reliably triggered by myoclonic movements, this may involve sensory feedback conveyed from S1 cortex to the MEC. Indeed, myoclonic movements reliably trigger, via sensory feedback, thalamo-cortical oscillatory bursts of activity in the S1 cortex of newborn rodents (Khazipov et al., 2004; An et al., 2014; Akhmetshina et al., 2016; Dooley et al., 2020). Since there is no direct input from S1 to the MEC, a further transmission of sensory feedback from S1 to the MEC should involve some relay areas (Witter et al., 2017). These relay stations may include the perirhinal cortex (Burwell and Amaral, 1998), the retrosplenial cortex (Sugar and Witter, 2016), and the postrhinal cortex (Lagartos-Donate et al., 2022); noteworthy, the latter two areas already establish functional inputs to MEC during the first postnatal week. Alternatively, the link between MEC-SPs and spontaneous myoclonic movements may be supported by a non-canonical reticulo-limbic circuit via the septum, which is activated during startles (Zhang et al., 2018), consistent with a triggering role of the septum in the generation of cortical waves in cultured coronal slices *in vitro* during the first postnatal week (Conhaim et al., 2011). Generation of MEC-SPs may also involve local MEC connections including recurrent and deep to superficial synapses (Quilichini et al., 2010; Zhang et al., 2014; Witter et al., 2017; Rozov et al., 2020). Moreover, these local connections are important as evidenced by the presence of spontaneous activity, very similar to MEC-SPs *in vivo*, in the isolated entorhinal-hippocampal slices *in vitro*, and their persistence after surgical severing of connections with the hippocampus (Sheroziya et al., 2009; Namiki et al., 2013; Unichenko et al., 2015). These observations also suggest that the generation of MEC-SPs primarily involves local circuitry, whereas sensory feedback from movements plays only a triggering role in coupling MEC-SPs (and eSPWs) to movements. This is further supported by the persistence of MEC-driven eSPWs in the hippocampus of immobilized neonatal rats under general anesthesia (Leinekugel et al., 2002; Gainutdinov et al., 2023). While previous studies emphasized the pivotal role of spontaneously bursting L3 neurons in the generation of MEC-SPs in neonatal rodent MEC slices *in vitro* (Sheroziya et al., 2009; Namiki et al., 2013; Unichenko et al., 2015), here we observed that L5 neurons fire before L3 neurons during MEC-SPs *in vivo*. These observations are consistent with the highest excitability of L5 neurons, their high propensity for spontaneous firing, and with the leading role of deep layers in self-generated cortical activity such as the UP-states of slow cortical oscillations (Sanchez-Vives and Mccormick, 2000; Isomura et al., 2006; Sakata and Harris, 2009; Reyes-Puerta et al., 2015; Senzai et al., 2019). Of note, despite of the limited involvement of CA1 and subicular inputs in initiation of MEC-SPs, these connections are in place during the first postnatal week (Khalilov et al., 1999; Canto et al., 2019), and their activation during eSPWs may contribute to the late phase of population burst in MEC.

Our main finding is that neurons in deep and superficial MEC layers are activated synchronously during MEC-SPs, and that neuronal firing in both superficial and deep MEC layers precedes the activation of CA1 neurons during eSPWs. This is remarkably different from the spatiotemporal dynamics in the entorhinal-hippocampal system during SPW-Rs in the adult brain. Indeed, during adult SPW-Rs, deep MEC neurons are activated following CA1 pyramidal cells by direct monosynaptic inputs from CA1 pyramidal cells or via intermediate activation of subicular neurons, whereas neurons in superficial MEC layers are weakly modulated by SPW-Rs (Chrobak and Buzsaki, 1994, 1996; Isomura et al., 2006; Roth et al., 2016; Rozov et al., 2020). Thus, the sequence of activation of hippocampal CA1 neurons and deep MEC neurons during sharp waves changes during development from a lead of deep MEC neurons during eSPWs in neonates to a lead of CA1 neurons during adult SPW-Rs. This provides further evidence for a difference in the generative mechanisms of eSPWs versus adult SPW-Rs, despite a similarity in the electrophysiological traits of these two distinct activity patterns, and supports the transition in understanding from eSPWs as immature prototypes of adult SPWs to a bottom-up model of eSPW genesis, driven primarily by inputs from the entorhinal cortex, marking a significant paradigm shift in entorhinal-hippocampal circuitry dynamics during development. Initially, eSPWs were viewed as nascent forms of SPWs, suggesting that eSPWs emerged spontaneously within the hippocampal circuitry and represented early manifestations of the network dynamics observed in adult hippocampal function. However, recent studies and present work challenge this traditional view, proposing a bottom-up model wherein eSPWs are predominantly initiated by inputs originating from the entorhinal cortex. In this revised framework, eSPWs are seen as arising from the orchestrated interplay between the entorhinal cortex and hippocampal circuitry, with inputs from the former triggering and shaping the dynamics of the latter. This perspective emphasizes the significance of sensory inputs, particularly somatosensory feedback from myoclonic movements, in driving hippocampal network activity during development to support the activity-dependent formation of the entorhinal-hippocampal network. Our study also suggests that during eSPWs, there is a limited relay of information from the CA1 hippocampus to the deep layers of the MEC during eSPWs, in contrast to adult SPW-Rs. Adult SPW-Rs are known to support the transfer of transiently stored hippocampal information to long-term engrams in neocortical networks, contributing to memory consolidation (Buzsaki, 1986; Siapas and Wilson, 1998; Girardeau et al., 2009; Nakashiba et al., 2009; Buzsaki, 2015; Squire et al., 2015). This limited communication between CA1 and the deep MEC in newborns may contribute to delayed development of the hippocampal-dependent memory and infantile amnesia (Baram et al., 2019). Our study also raises questions for future research on the developmental stage at which the change in the temporal dynamics of neuronal activation in CA1 and EC occurs, and on the potential mechanisms and functional implications of the developmental change in the sequence of CA1 and EC activation during sharp waves, which, according to recent studies, may involve the development of inhibitory circuitry (Dard et al., 2022; Pochinok et al., 2024).

## Data availability statement

The raw data supporting the conclusions of this article will be made available by the authors, without undue reservation.

## Ethics statement

The animal study was approved by French National Institute of Health and Medical Research (APAFIS #16992-2020070612319346 v2) and the Local Ethical Committee of Kazan Federal University (#24/22.09.2020). The study was conducted in accordance with the local legislation and institutional requirements.

## Author contributions

DS: Validation, Writing – review & editing, Data curation, Formal analysis, Investigation, Methodology, Visualization. AN: Data curation, Formal analysis, Investigation, Methodology, Validation, Visualization, Writing – review & editing, Software. RK: Validation, Writing – review & editing, Conceptualization, Writing – original draft. GV: Conceptualization, Data curation, Formal analysis, Funding acquisition, Investigation, Methodology, Project administration, Resources, Software, Supervision, Validation, Visualization, Writing – original draft, Writing – review & editing.

## References

[ref1] Akhmetshina D. Nasretdinov A. Zakharov A. Valeeva G. Khazipov R. (2016). The nature of the sensory input to the neonatal rat barrel cortex. J. Neurosci. 36, 9922–9932. doi: 10.1523/JNEUROSCI.1781-16.2016, PMID: 27656029 PMC6705573

[ref2] An S. M. Kilb W. Luhmann H. J. (2014). Sensory-evoked and spontaneous gamma and spindle bursts in neonatal rat motor cortex. J. Neurosci. 34, 10870–10883. doi: 10.1523/JNEUROSCI.4539-13.2014, PMID: 25122889 PMC6705262

[ref3] Baram T. Z. Donato F. Holmes G. L. (2019). Construction and disruption of spatial memory networks during development. Learn. Mem. 26, 206–218. doi: 10.1101/lm.049239.118, PMID: 31209115 PMC6581006

[ref4] Bellistri E. Aguilar J. Brotons-Mas J. R. Foffani G. De La Prida L. M. (2013). Basic properties of somatosensory-evoked responses in the dorsal hippocampus of the rat. J. Physiol. 591, 2667–2686. doi: 10.1113/jphysiol.2013.251892, PMID: 23420661 PMC3678049

[ref5] Ben Ari Y. Gaiarsa J. L. Tyzio R. Khazipov R. (2007). GABA: a Pioneer transmitter that excites immature neurons and generates primitive oscillations. Physiol. Rev. 87, 1215–1284. doi: 10.1152/physrev.00017.2006, PMID: 17928584

[ref6] Ben-Ari Y. Cherubini E. Corradetti R. Gaïarsa J.-L. (1989). Giant synaptic potentials in immature rat CA3 hippocampal neurones. J. Physiol. Lond. 416, 303–325. doi: 10.1113/jphysiol.1989.sp017762, PMID: 2575165 PMC1189216

[ref7] Brankack J. Buzsaki G. (1986). Hippocampal responses evoked by tooth pulp and acoustic stimulation: depth profiles and effect of behavior. Brain Res. 378, 303–314. doi: 10.1016/0006-8993(86)90933-9, PMID: 3730880

[ref8] Buhl D. L. Buzsaki G. (2005). Developmental emergence of hippocampal fast-field "ripple" oscillations in the behaving rat pups. Neuroscience 134, 1423–1430. doi: 10.1016/j.neuroscience.2005.05.030, PMID: 16039793 PMC1851000

[ref9] Burwell R. D. Amaral D. G. (1998). Perirhinal and postrhinal cortices of the rat: interconnectivity and connections with the entorhinal cortex. J. Comp. Neurol. 391, 293–321. doi: 10.1002/(SICI)1096-9861(19980216)391:3<293::AID-CNE2>3.0.CO;2-X 9492202

[ref10] Buzsaki G. (1986). Hippocampal sharp waves: their origin and significance. Brain Res. 398, 242–252. doi: 10.1016/0006-8993(86)91483-6, PMID: 3026567

[ref11] Buzsaki G. (2015). Hippocampal sharp wave-ripple: a cognitive biomarker for episodic memory and planning. Hippocampus 25, 1073–1188. doi: 10.1002/hipo.22488, PMID: 26135716 PMC4648295

[ref12] Canto C. B. Koganezawa N. Lagartos-Donate M. J. O'reilly K. C. Mansvelder H. D. Witter M. P. (2019). Postnatal development of functional projections from Parasubiculum and Presubiculum to medial entorhinal cortex in the rat. J. Neurosci. 39, 8645–8663. doi: 10.1523/JNEUROSCI.1623-19.2019 31511428 PMC6820215

[ref13] Canto C. B. Wouterlood F. G. Witter M. P. (2008). What does the anatomical Organization of the Entorhinal Cortex Tell us? Neural Plast. 2008:381243, 1–18. doi: 10.1155/2008/381243 18769556 PMC2526269

[ref14] Chrobak J. J. Buzsaki G. (1994). Selective activation of deep layer (V-VI) retrohippocampal cortical neurons during hippocampal sharp waves in the behaving rat. J. Neurosci. 14, 6160–6170. doi: 10.1523/JNEUROSCI.14-10-06160.1994, PMID: 7931570 PMC6576977

[ref15] Chrobak J. J. Buzsaki G. (1996). High-frequency oscillations in the output networks of the hippocampal-entorhinal axis of the freely behaving rat. J. Neurosci. 16, 3056–3066. doi: 10.1523/JNEUROSCI.16-09-03056.1996, PMID: 8622135 PMC6579047

[ref16] Conhaim J. Easton C. R. Becker M. I. Barahimi M. Cedarbaum E. R. Moore J. G.. (2011). Developmental changes in propagation patterns and transmitter dependence of waves of spontaneous activity in the mouse cerebral cortex. J. Physiol. 589, 2529–2541. doi: 10.1113/jphysiol.2010.202382, PMID: 21486817 PMC3115823

[ref17] Cossart R. Khazipov R. (2022). How development sculpts hippocampal circuits and function. Physiol. Rev. 102, 343–378. doi: 10.1152/physrev.00044.2020, PMID: 34280053

[ref18] Dard R. F. Leprince E. Denis J. Rao B. S. Suchkov D. Boyce R.. (2022). The rapid developmental rise of somatic inhibition disengages hippocampal dynamics from self-motion. eLife 11:e78116. doi: 10.7554/eLife.78116, PMID: 35856497 PMC9363116

[ref19] Donato F. Jacobsen R. I. Moser M. B. Moser E. I. (2017). Stellate cells drive maturation of the entorhinal-hippocampal circuit. Science 355:eaai8178. doi: 10.1126/science.aai8178, PMID: 28154241

[ref20] Dooley J. C. Glanz R. M. Sokoloff G. Blumberg M. S. (2020). Self-generated whisker movements drive state-dependent sensory input to developing barrel cortex. Curr. Biol. 30, 2404–2410.e4. doi: 10.1016/j.cub.2020.04.045, PMID: 32413304 PMC7314650

[ref21] Freeman J. A. Nicholson C. (1975). Experimental optimization of current source-density technique for anuran cerebellum. J. Neurophysiol. 38, 369–382. doi: 10.1152/jn.1975.38.2.369, PMID: 165272

[ref22] Gainutdinov A. Shipkov D. Sintsov M. Fabrizi L. Nasretdinov A. Khazipov R.. (2023). Somatosensory-evoked early sharp waves in the neonatal rat hippocampus. Int. J. Mol. Sci. 24:8721. doi: 10.3390/ijms24108721, PMID: 37240066 PMC10217913

[ref23] Girardeau G. Benchenane K. Wiener S. I. Buzsaki G. Zugaro M. B. (2009). Selective suppression of hippocampal ripples impairs spatial memory. Nat. Neurosci. 12, 1222–1223. doi: 10.1038/nn.2384, PMID: 19749750

[ref24] Graf J. Zhang C. Marguet S. L. Herrmann T. Flossmann T. Hinsch R.. (2021). A limited role of NKCC1 in telencephalic glutamatergic neurons for developing hippocampal network dynamics and behavior. Proc. Natl. Acad. Sci. U. S. A 118:e2014784118. doi: 10.1073/pnas.2014784118 33782119 PMC8040628

[ref25] Griguoli M. Cherubini E. (2017). Early correlated network activity in the Hippocampus: its putative role in shaping neuronal circuits. Front. Cell. Neurosci. 11:255. doi: 10.3389/fncel.2017.00255, PMID: 28878628 PMC5572250

[ref26] Isomura Y. Sirota A. Ozen S. Montgomery S. Mizuseki K. Henze D. A.. (2006). Integration and segregation of activity in entorhinal-hippocampal subregions by neocortical slow oscillations. Neuron 52, 871–882. doi: 10.1016/j.neuron.2006.10.023, PMID: 17145507

[ref27] Karlsson K. A. Mohns E. J. Di Prisco G. V. Blumberg M. S. (2006). On the co-occurrence of startles and hippocampal sharp waves in newborn rats. Hippocampus 16, 959–965. doi: 10.1002/hipo.20224, PMID: 17009334 PMC2645543

[ref28] Khalilov I. Dzhala V. Medina I. Leinekugel X. Melyan Z. Lamsa K.. (1999). Maturation of kainate-induced epileptiform activities in interconnected intact neonatal limbic structures *in vitro*. Eur. J. Neurosci. 11, 3468–3480. doi: 10.1046/j.1460-9568.1999.00768.x, PMID: 10564355

[ref29] Khazipov R. Sirota A. Leinekugel X. Holmes G. L. Ben Ari Y. Buzsaki G. (2004). Early motor activity drives spindle bursts in the developing somatosensory cortex. Nature 432, 758–761. doi: 10.1038/nature03132, PMID: 15592414

[ref30] Khazipov R. Zaynutdinova D. Ogievetsky E. Valeeva G. Mitrukhina O. Manent J. B.. (2015). Atlas of the postnatal rat brain in stereotaxic coordinates. Front. Neuroanat. 9:110.3389/fnana.2015.00161. doi: 10.3389/fnana.2015.00161 26778970 PMC4688355

[ref31] Lagartos-Donate M. J. Doan T. P. Girao P. J. B. Witter M. P. (2022). Postnatal development of projections of the postrhinal cortex to the entorhinal cortex in the rat. eNeuro 9, ENEURO.0057–ENEU22.2022. doi: 10.1523/ENEURO.0057-22.2022, PMID: 35715208 PMC9239852

[ref32] Leinekugel X. Khazipov R. Cannon R. Hirase H. Ben Ari Y. Buzsaki G. (2002). Correlated bursts of activity in the neonatal hippocampus *in vivo*. Science 296, 2049–2052. doi: 10.1126/science.1071111, PMID: 12065842

[ref33] Marguet S. L. Le-Schulte V. T. Merseburg A. Neu A. Eichler R. Jakovcevski I.. (2015). Treatment during a vulnerable developmental period rescues a genetic epilepsy. Nat. Med. 21, 1436–1444. doi: 10.1038/nm.3987, PMID: 26594844

[ref34] Mohns E. J. Blumberg M. S. (2010). Neocortical activation of the hippocampus during sleep in infant rats. J. Neurosci. 30, 3438–3449. doi: 10.1523/JNEUROSCI.4832-09.2010, PMID: 20203203 PMC2851014

[ref35] Mohns E. J. Karlsson K. A. Blumberg M. S. (2007). Developmental emergence of transient and persistent hippocampal events and oscillations and their association with infant seizure susceptibility. Eur. J. Neurosci. 26, 2719–2730. doi: 10.1111/j.1460-9568.2007.05928.x, PMID: 17973923 PMC2556895

[ref36] Murata Y. Colonnese M. T. (2020). GABAergic interneurons excite neonatal hippocampus *in vivo*. Sci. Adv. 6:eaba1430. doi: 10.1126/sciadv.aba1430, PMID: 32582852 PMC7292633

[ref37] Nakashiba T. Buhl D. L. Mchugh T. J. Tonegawa S. (2009). Hippocampal CA3 output is crucial for ripple-associated reactivation and consolidation of memory. Neuron 62, 781–787. doi: 10.1016/j.neuron.2009.05.013, PMID: 19555647 PMC2728553

[ref38] Namiki S. Norimoto H. Kobayashi C. Nakatani K. Matsuki N. Ikegaya Y. (2013). Layer III neurons control synchronized waves in the immature cerebral cortex. J. Neurosci. 33, 987–1001. doi: 10.1523/JNEUROSCI.2522-12.2013, PMID: 23325237 PMC6704853

[ref39] Pochinok I. Stober T. M. Triesch J. Chini M. Hanganu-Opatz I. L. (2024). A developmental increase of inhibition promotes the emergence of hippocampal ripples. Nat. Commun. 15:738. doi: 10.1038/s41467-024-44983-z 38272901 PMC10810866

[ref40] Quilichini P. Sirota A. Buzsaki G. (2010). Intrinsic circuit organization and theta-gamma oscillation dynamics in the entorhinal cortex of the rat. J. Neurosci. 30, 11128–11142. doi: 10.1523/JNEUROSCI.1327-10.2010, PMID: 20720120 PMC2937273

[ref41] Reyes-Puerta V. Sun J. J. Kim S. Kilb W. Luhmann H. J. (2015). Laminar and columnar structure of sensory-evoked multineuronal spike sequences in adult rat barrel cortex *in vivo*. Cereb. Cortex 25, 2001–2021. doi: 10.1093/cercor/bhu007, PMID: 24518757

[ref42] Roth F. C. Beyer K. M. Both M. Draguhn A. Egorov A. V. (2016). Downstream effects of hippocampal sharp wave ripple oscillations on medial entorhinal cortex layer V neurons *in vitro*. Hippocampus 26, 1493–1508. doi: 10.1002/hipo.22623, PMID: 27479916

[ref43] Rozov A. Rannap M. Lorenz F. Nasretdinov A. Draguhn A. Egorov A. V. (2020). Processing of hippocampal network activity in the receiver network of the medial entorhinal cortex layer V. J. Neurosci. 40, 8413–8425. doi: 10.1523/JNEUROSCI.0586-20.2020, PMID: 32978288 PMC7605420

[ref44] Sakata S. Harris K. D. (2009). Laminar structure of spontaneous and sensory-evoked population activity in auditory cortex. Neuron 64, 404–418. doi: 10.1016/j.neuron.2009.09.020 19914188 PMC2778614

[ref45] Sanchez-Vives M. V. Mccormick D. A. (2000). Cellular and network mechanisms of rhythmic recurrent activity in neocortex. Nat. Neurosci. 3, 1027–1034. doi: 10.1038/79848, PMID: 11017176

[ref46] Senzai Y. Fernandez-Ruiz A. Buzsaki G. (2019). Layer-specific physiological features and Interlaminar interactions in the primary visual cortex of the mouse. Neuron 101, 500–513.e5. doi: 10.1016/j.neuron.2018.12.009, PMID: 30635232 PMC6367010

[ref47] Sheroziya M. G. Von Bohlen Und H. O. Unsicker K. Egorov A. V. (2009). Spontaneous bursting activity in the developing entorhinal cortex. J. Neurosci. 29, 12131–12144. doi: 10.1523/JNEUROSCI.1333-09.2009, PMID: 19793971 PMC6666150

[ref48] Siapas A. G. Wilson M. A. (1998). Coordinated interactions between hippocampal ripples and cortical spindles during slow-wave sleep. Neuron 21, 1123–1128. doi: 10.1016/S0896-6273(00)80629-7, PMID: 9856467

[ref49] Squire L. R. Genzel L. Wixted J. T. Morris R. G. (2015). Memory consolidation. Cold Spring Harb. Perspect. Biol. 7:a021766. doi: 10.1101/cshperspect.a021766, PMID: 26238360 PMC4526749

[ref50] Sugar J. Witter M. P. (2016). Postnatal development of retrosplenial projections to the parahippocampal region of the rat. eLife 5:e13925. doi: 10.7554/eLife.13925, PMID: 27008178 PMC4859804

[ref51] Unichenko P. Yang J. W. Luhmann H. J. Kirischuk S. (2015). Glutamatergic system controls synchronization of spontaneous neuronal activity in the murine neonatal entorhinal cortex. Pflugers Arch. 467, 1565–1575. doi: 10.1007/s00424-014-1600-5 25163767

[ref52] Valeeva G. Janackova S. Nasretdinov A. Rychkova V. Makarov R. Holmes G. L.. (2019a). Emergence of coordinated activity in the developing entorhinal-hippocampal network. Cereb. Cortex 29, 906–920. doi: 10.1093/cercor/bhy309, PMID: 30535003 PMC6319314

[ref53] Valeeva G. Nasretdinov A. Rychkova V. Khazipov R. (2019b). Bilateral synchronization of hippocampal early sharp waves in neonatal rats. Front. Cell. Neurosci. 13:29. doi: 10.3389/fncel.2019.00029, PMID: 30792630 PMC6374346

[ref54] Valeeva G. Rychkova V. Vinokurova D. Nasretdinov A. Khazipov R. (2020). Early sharp wave synchronization along the septo-temporal axis of the neonatal rat hippocampus. Zhurnal Vysshei Nervnoi Deyatelnosti Imeni I P Pavlova 70, 341–350. doi: 10.31857/S0044467720030132

[ref55] Witter M. P. Doan T. P. Jacobsen B. Nilssen E. S. Ohara S. (2017). Architecture of the entorhinal cortex a review of entorhinal anatomy in rodents with some comparative notes. Front. Syst. Neurosci. 11:46. doi: 10.3389/fnsys.2017.00046 28701931 PMC5488372

[ref56] Zhang G. W. Sun W. J. Zingg B. Shen L. He J. Xiong Y.. (2018). A non-canonical reticular-limbic central auditory pathway via medial septum contributes to fear conditioning. Neuron 97, 406–417.e4. doi: 10.1016/j.neuron.2017.12.010, PMID: 29290554 PMC5798467

[ref57] Zhang S. J. Ye J. Couey J. J. Witter M. Moser E. I. Moser M. B. (2014). Functional connectivity of the entorhinal - hippocampal space circuit. Philos. Trans. R. Soc. B Biol. Sci. 369:20120516. doi: 10.1098/rstb.2012.0516, PMID: 24366130 PMC3866440

