# Supplementary material for: Synchronous excitation in the superficial and deep layers of the medial entorhinal cortex precedes early sharp waves in the neonatal rat hippocampus
Source: Front Cell Neurosci. 2024 Apr 26;18:1403073. doi: 10.3389/fncel.2024.1403073 (PMC11082381; doi:10.3389/fncel.2024.1403073)
Supplement: Supplementary file 1 [file Data_Shee_1.docx]

Supplementary Material

## 1. Supplementary Figures


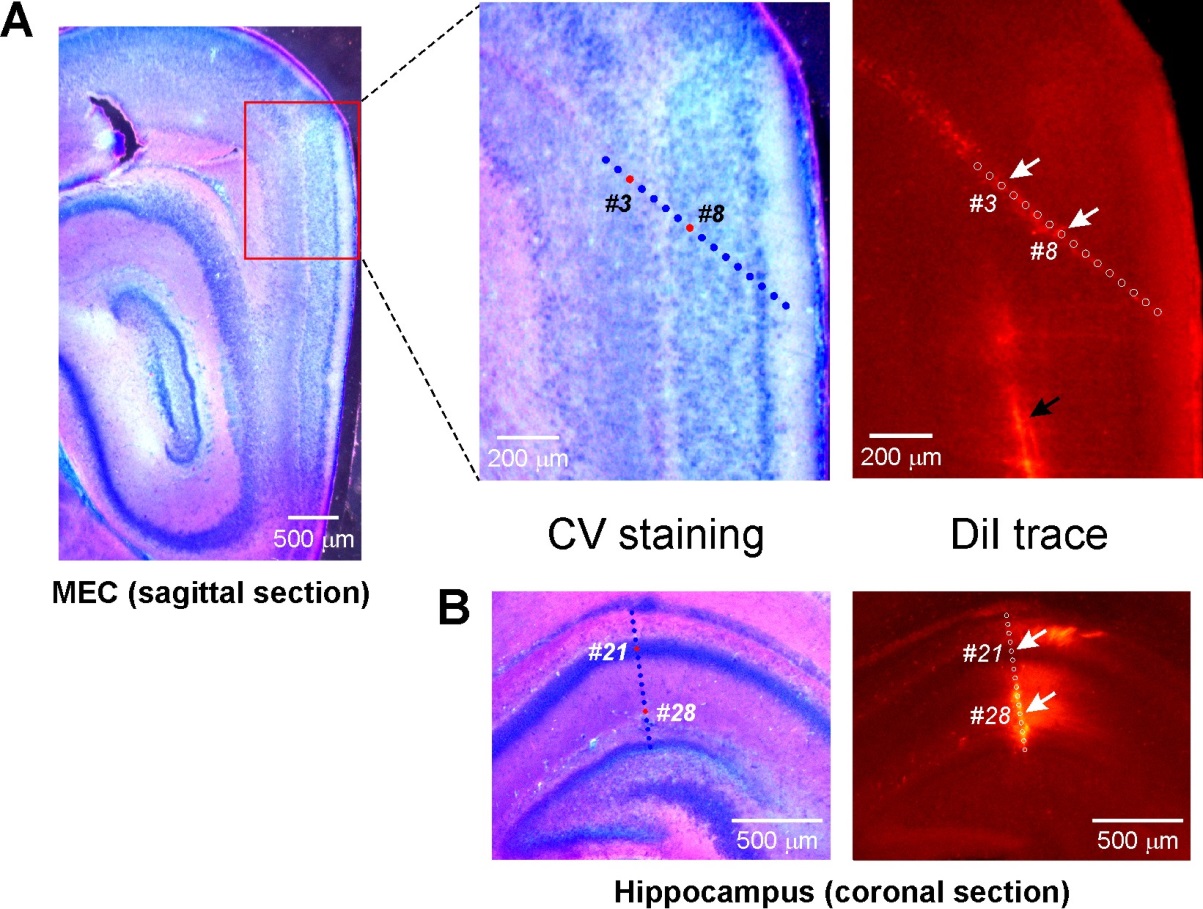


**Supplementary Figure 1. An identification of silicone probe location by DiI track in the MEC and hippocampus presented in Fig. 1A.** Left, the microphotographs of 100 μm-thick (**A**) sagittal MEC slice and (**B**) coronal hippocampal slice after cresyl violet staining, with overlaid silicone probe recording sites. Right, the microphotographs of corresponding slices with a DiI track left by silicone probe in the brain tissue. Example recording traces from sites #3, #8, #21 and #28 (marked by red color on the left panels and by white arrows on the right panels) were presented in Figure 1B,C. A black arrow on panel A (left) points to DiI track showing a probe location during the recording made along MEC layers (animal #8 in Supplementary Figure 3).


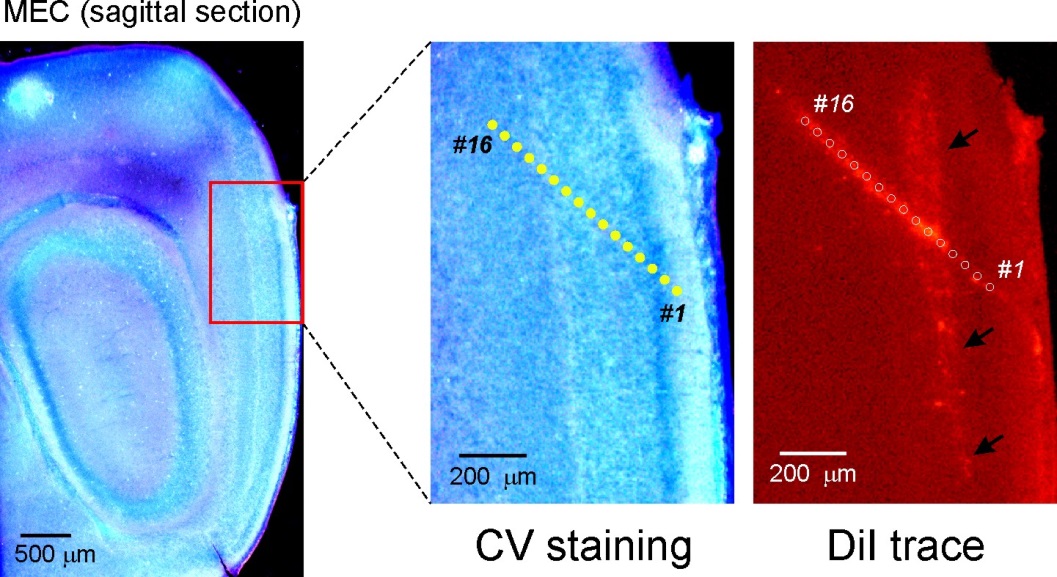


**Supplementary Figure 2. An identification of silicone probe location by DiI track in the MEC presented in Fig. 3A.** Left, the microphotograph of 100 μm-thick sagittal MEC slice with overlaid silicone probe recording sites (#1-16). Right, the microphotograph of the same slices with a DiI track of the silicone probe. LFP traces from the probe sites 1-16 were presented in Figure 3B. Black arrows point to another DiI track showing the probe location during the recording made along MEC layers (animal #5, lower trace in Supplementary Figure 3).


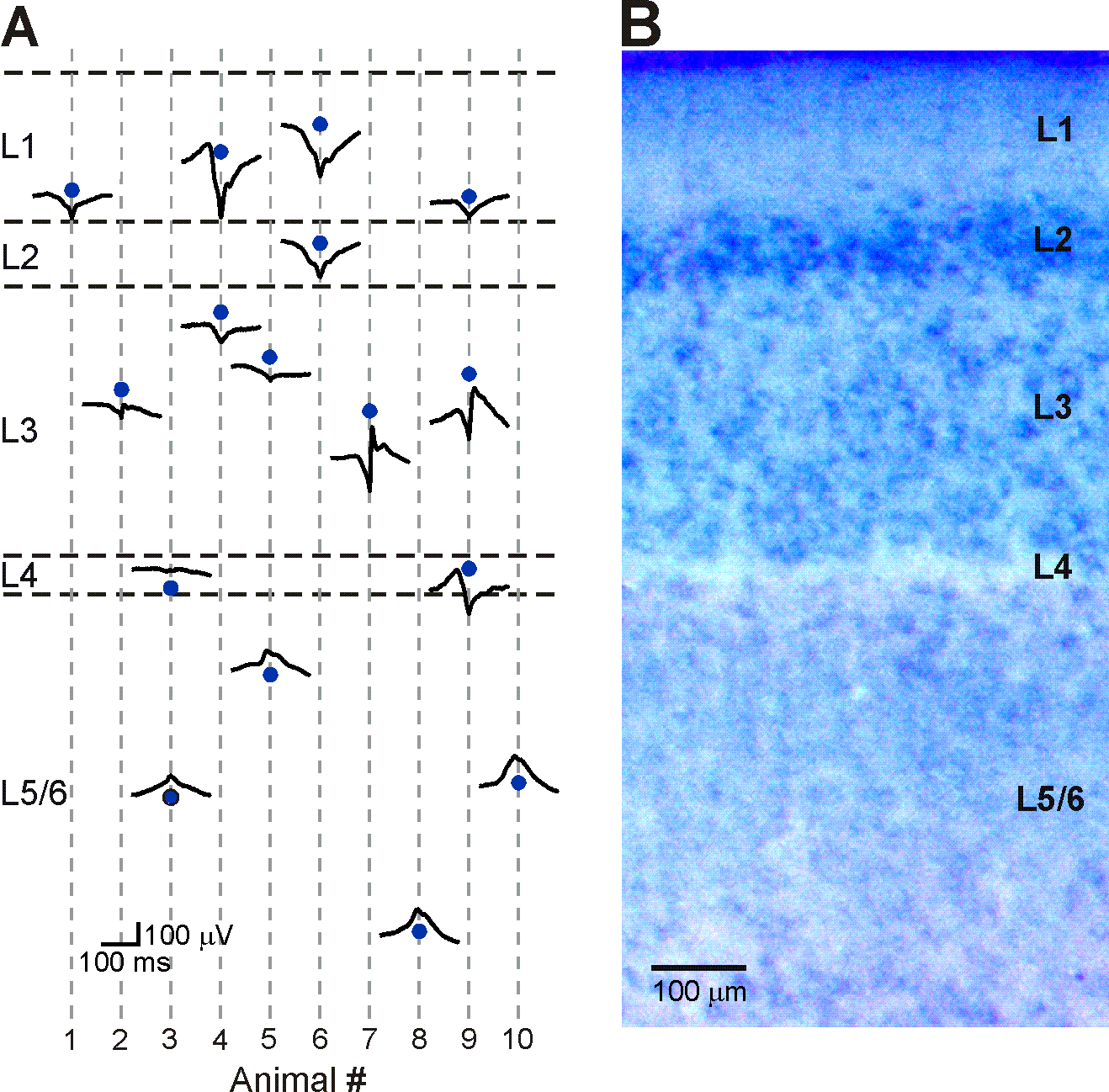


**Supplementary Figure 3.** **LFP depth profiles of the MEC sharp potential during recordings with silicone probes located along the MEC layers. (A)** Averaged LFP waveforms of MEC sharp potentials recorded in different cortical layers from ten P4-7 rats. From one to three electrode insertions were made in each animal. MEC-SPs from a single recording site are shown for each insertion. The relative depth of recording sites (blue circles) was normalized to the distance between L1/L2 and L3/L4 borders. (**B**) The microphotograph of 100 μm-thick MEC sagittal section of a P7 rat (cresyl violet staining).

# 2. Supplementary Tables

| MUA PETH peak value | **Median** | **Q1** | **Q3** | **N rats** | **P value** |
| --- | --- | --- | --- | --- | --- |
| **MEC L3 vs eSPW** | 11.3 | 5.4 | 14.1 | 17 | 2.9 × 10^-4^ |
| **MEC L5 vs eSPW** | 11.0 | 6.2 | 13.6 | 17 | 2.9 × 10^-4^ |
| **CA1 pcl vs eSPW** | 9.1 | 6.2 | 12.4 | 17 | 2.9 × 10^-4^ |

**Supplementary Table 1.** Statistical data (presented in Figure 2A,B) on PETH peak value of normalized MUA frequency in MEC L3, MEC L5 and CA1 pcl within [-100; +100] ms time window around the eSPW peak. P value shows the significance of MUA frequency increase during eSPWs compared to baseline frequency. A significant difference (p<0.05, Wilcoxon signed rank test) is indicated in green.

| MUA PETH peak time, **ms** | **Median** | **Q1** | **Q3** | **N**  **eSPWs** | **N**  **APs** | **N rats** | **P value,**  **L3 - L5** | **P value,**  **MEC L5 - CA1 pcl** | **P value,**  **MEC L3 - CA1 pcl** |
| --- | --- | --- | --- | --- | --- | --- | --- | --- | --- |
| **MEC L3** | -1 | -6.0 | 5.0 | 1196 | 4915 | 17 | 0.5809 |  | 7.3 × 10^-6^ |
| **MEC L5** | -2 | -5.5 | 3.3 | 1196 | 5408 | 17 | 0.5809 | 5.7 × 10 ^-6^ |  |
| **CA1 pcl** | 17 | 14.5 | 21.3 | 1196 | 6779 | 17 |  | 5.7 × 10^-6^ | 7.3 × 10^-6^ |

**Supplementary Table 2**. Statistical data (presented in Figure 2C) on MUA PETH peak time in MEC L3, MEC L5 and CA1 pcl calculated within [-100; +100] ms relative to the eSPW peak. A non-significant difference between groups (p > 0.05) is highlighted in red and a significant difference (p < 0.05) - in green, Wilcoxon rank sum test.

| MUA cross-corr. peak value, APs per ms | **Median** | **Q1** | **Q3** | **N rats** |
| --- | --- | --- | --- | --- |
| **MEC L3 vs CA1 pcl** | 0.04 | 0.02 | 0.06 | 17 |
| **MEC L5 vs CA1 pcl** | 0.05 | 0.03 | 0.07 | 17 |
| **MEC L5 vs L3** | 0.05 | 0.03 | 0.07 | 17 |

**Supplementary Table 3.** Group data (presented in Figure 2D,E) on the cross-correlation peak value of MUA recorded in MEC L3, MEC L5 and CA1 pcl within [-50; +100] ms relative to the eSPW peak.

| MUA cross-corr. peak time lag, ms | **Median** | **Q1** | **Q3** | **N**  **eSPWs** | **N**  **APs** | **N rats** | **P value** |
| --- | --- | --- | --- | --- | --- | --- | --- |
| **MEC L3 vs CA1 pcl** | -14 | -22 | -7 | 1196 | 4523 (L3) 6314 (CA1) | 17 | 0.0437 |
| **MEC L5 vs CA1 pcl** | -24 | -26 | -14 | 1196 | 4955 (L5)  6314 (CA1) | 17 |  |
| **MEC L5 vs L3** | -6 | -10 | -4 | 1196 | 4955 (L5)  4523 (L3) | 17 | 0.0006 |

**Supplementary Table 4.** Statistical data (presented in Figure 2F) on the delay between cross-correlation peak times of MUA recorded in MEC L3, MEC L5 and CA1 pcl and detected within [-50; +100] ms relative to the eSPW peak. A significant difference between groups (p<0.05, Wilcoxon rank sum test) is indicated in green.

| Sink depth, μm | **Median** | **Q1** | **Q3** | **N**  **eSPWs** | **N**  **rats** | **P value,**  **Sink 1 – Sink 2** | **P value,**  **Sink 2 – Sink 3** | **P value,**  **Sink 1 – Sink 3** |
| --- | --- | --- | --- | --- | --- | --- | --- | --- |
| **Sink 1** | 298 | 281 | 330 | 1271 | 18 | 9.0 × 10 ^-5^ |  | 4.8 × 10 ^-7^ |
| **Sink 2** | 203 | 181 | 219 | 615 | 8 | 9.0 × 10 ^-5^ | 5.3 × 10 ^-4^ |  |
| **Sink 3** | 142 | 123 | 160 | 1189 | 17 |  | 5.3 × 10 ^-4^ | 4.8 × 10 ^-7^ |

**Supplementary Table 5.** Statistical data (presented in Figure 3D) on MEC sharp potential-associated current sink location relative to MEC L3/L4 border. A significant difference between groups (p<0.05, Wilcoxon rank sum test) is indicated in green.

| Normalized sink depth | **Median** | **Q1** | **Q3** | **N**  **eSPWs** | **N rats** |
| --- | --- | --- | --- | --- | --- |
| **Sink 1** | 1.18 | 1.07 | 1.31 | 1271 | 18 |
| **Sink 2** | 0.80 | 0.73 | 0.86 | 615 | 8 |
| **Sink 3** | 0.56 | 0.46 | 0.63 | 1189 | 17 |

**Supplementary Table 6.** Group data (presented in Figure 3E) on MEC sharp potential-associated current sink depth normalized to the distance between MEC L3/4 and L2/3 borders.

| Sink amplitude, a.u. | **Median** | **Q1** | **Q3** | **N**  **eSPWs** | **N rats** | **P value,**  **S1 - S2** | **P value,**  **S2 - S3** | **P value,**  **S1 - S3** |
| --- | --- | --- | --- | --- | --- | --- | --- | --- |
| **Sink 1** | 34.98 | 25.10 | 60.34 | 1271 | 18 | 0.00034 |  | 0.00003 |
| **Sink 2** | 12.97 | 8.56 | 19.81 | 615 | 8 | 0.00034 | 0.46650 |  |
| **Sink 3** | 15.29 | 10.29 | 21.59 | 1189 | 17 |  | 0.46650 | 0.00003 |

**Supplementary Table 7.** Statistical data (presented in Figure 3F) on the amplitude of current sinks associated with MEC sharp potentials. A non-significant difference between groups (p > 0.05) is highlighted in red and a significant difference (p < 0.05) - in green, Wilcoxon rank sum test.
